# Vitexin as an active ingredient in passion flower with potential as an agent for nicotine cessation: vitexin antagonism of the expression of nicotine locomotor sensitization in rats

**DOI:** 10.1080/13880209.2018.1561725

**Published:** 2019-02-01

**Authors:** Samantha Bedell, Jacob Wells, Qinfeng Liu, Chris Breivogel

**Affiliations:** Department of Pharmaceutical Sciences, Campbell University College of Pharmacy & Health Sciences, Buies Creek, NC, USA

**Keywords:** Addiction, flavonoid, natural product, smoking cessation, *Passiflora incarnata*

## Abstract

**Context:** Nicotine, a bioactive component of tobacco, is highly addictive. Numerous therapies have been developed for smoking cessation, and all have met with limited success. Our laboratory has previously shown that an extract of *Passiflora incarnata* Linn. (Passifloraceae) antagonized the expression of nicotine locomotor sensitization in rats.

**Objective:** This study examined the ability of vitexin, a flavonoid found in *P. incarnata,* to ameliorate the signs of nicotine sensitization in rats.

**Materials and methods:** Male Wistar rats were administered 0.4 mg/kg nicotine or vehicle (*n* = 16–18 per group) once a day for four consecutive days. Nicotine administration produces sensitization of locomotor activity. On the fifth day, locomotor activity was monitored as rats from each treatment group were administered either 30 or 60 mg/kg vitexin or its vehicle (*n* = 4–6 per group) 30 min before a challenge dose of 0.4 mg/kg nicotine.

**Results:** The challenge dose of nicotine resulted in locomotor activity in rats sensitized to nicotine for 4 days that was approximately twice that measured in rats treated with vehicle during the sensitization phase. Rats sensitized to nicotine and then treated with 60 mg/kg vitexin prior to the nicotine challenge exhibited a level of locomotor activity equivalent to the vehicle-treated controls.

**Discussion:** Vitexin antagonized the expression of nicotine locomotor sensitization in rats as the whole extract did in the previous study.

**Conclusion:** Vitexin should be examined in future studies to evaluate its potential for treating nicotine addiction in humans.

## Introduction

Tobacco is the single greatest cause of preventable disease and premature death in the United States today (Moeller and Sun 2010). Nicotine, the psychoactive ingredient, is both excitatory and addictive, which account for its stimulating, yet calming effects (Wadgave and Nagesh 2016). The compound binds to central nicotinic acetylcholine receptors causing release of the neurotransmitter dopamine in the mesolimbic area, the corpus striatum and the frontal cortex (Benowitz 1996). An increase in dopamine levels in the nucleus accumbens has been shown to be related to the addictive properties of nicotine (Dani and De Biasi 2001). No existing cessation pharmacotherapy including bupropion, varenicline or nicotine replacement is effective for more than about one-third of patients (Cahill et al. 2014).

Preparations of passion flower [*Passiflora incarnata* Linn. (Passifloraceae)] have been used in traditional and herbal medicines to treat anxiety, insomnia and seizures, but controlled clinical studies of passion flower extract for these indications are limited (Werneke et al. 2006). Passion flower extract has been examined in double-blind studies and shown to treat generalized anxiety disorder similar to oxazepram (Akhondzadeh et al. 2001), to help patients through opiate withdrawal (Akhondzadeh et al. 2001), and to alleviate pre-surgical anxiety (Movafegh et al. 2008). In laboratory rodents, passion flower extract or single chemical constituents of passion flower have been shown to be sedative and anxiolytic (Soulimani et al. 1997; Zanoli et al. 2000; Krenn 2002) or anticonvulsant (Nassiri-Asl et al. 2007). Moreover, several studies in laboratory rodents have demonstrated that whole extracts or an undisclosed benzoflavone moiety from passion flower are anxiolytic, antitussive and aphrodisiac (Dhawan et al. 2001, 2002). The same investigators (Dhawan et al. 2004) also showed that the benzoflavone compound, when given together with the addictive agent during the development of dependence, reduced the symptoms of antagonist-precipitated withdrawal to morphine, alcohol, nicotine, diazepam and Δ^9^-tetrahydrocannabinol.

Previous studies have demonstrated that an acute injection of nicotine to rats results in increased locomotion, and daily injections result in locomotor sensitization (Ksir et al. 1987; DiFranza and Wellman 2007; Li et al. 2008). This sensitization is believed to play an essential role in the development of addiction (Kayir et al. 2009). Screening for antagonism of the expression of nicotine locomotor sensitization is a validated method for identifying agents that may promote smoking cessation in humans (Kayir et al. 2009). A previous study by our laboratory showed that an extract of passion flower antagonized the expression of nicotine locomotor sensitization in rats (Breivogel and Jamerson 2012). The present study hypothesizes that vitexin is the component in the passion flower extract responsible for this activity.

## Materials and methods

### Solid-phase extraction (SPE) procedure for purifying passion flower extract

A 1 mL C18 SPE cartridge was pre-washed with 5 mL methanol followed by 5 mL of 0.1% formic acid in H_2_O. The commercial passion flower extract (0.3 mL; Nature’s Answer, Batch No: 132777) was diluted (×10) with deionized H_2_O and filtered through a 0.45 µm membrane before loading onto an SPE cartridge. The SPE was washed with 5 mL 0.1% formic acid in water, and then eluted with 2 mL 50:50 0.1% formic acid in water/methanol. The elution fraction was collected and analyzed using LC-MS.

### HPLC-mass spectrometry of passion flower extract

An Agilent 1260 HPLC coupled to an Agilent 6540B QTOF was used. An Agilent extended LC C18 column, 5 cm × 2.1 mm, 1.7 µm, was used. Gradient elution was performed with 95%/5% water, 0.1% formic acid:acetonitrile, 0.1% formic acid to 75% water, 0.1% formic acid: 25% acetonitrile, 0.1% formic acid from 0–16 min, then back to 5% acetonitrile, 0.1% formic acid from 16.1 to 19 min at a flow rate of 0.4 mL/min. Samples or standards were injected at 5 µL. The QTOF MS was equipped with a dual JetStream ESI probe at positive mode to provide a continuous injection of API-TOF Mass Reference (Agilent, Santa Clara, CA). It was tuned prior to HPLC-MS analysis to ensure mass error <10 ppm, and continuously calibrated with the reference to maintain mass accuracy during HPLC-MS analysis. The QTOF was run with the following settings: gas temperature 300 °C; drying gas flow rate 12 L/min; nebulizer pressure 35 psig; sheath gas temperature 325 °C; sheath gas flow rate 10 L/min; Vcap 4 kV; fragmentor 135 V; skimmer 65 V; OCT RF Vapp 750 V; mass scan range *m*/*z* 100–1000. High accuracy QTOF MS data were analyzed using MassHunter Qualitative Analysis B.06.00 software. Compound identification was processed by extracting MS ions with intensity above 20,000 counts and comparing with a user-defined database of reported plant flavonoids in literature for mass match with a mass defect tolerance of 0.05 Da.

### Drug treatment of rats

All animal work was approved by the Campbell University Institutional Animal Care and Use Committee (IACUC). Male Wistar rats, shipped from the Charles River facility in Morrisville, NC and housed in the Campbell University Animal facility, were provided with food and water *ad libitum* and kept on a 12 h light/dark cycle at approximately 21 °C for 2–3 weeks before beginning any treatments. For all experiments, rats were 45–65 days old and between 148 and 260 g. Prior to the treatments specified in each protocol below, each rat was drug-naïve. Injections of nicotine tartrate or its vehicle (water) were injected subcutaneously (s.c.) at 0.4 mg/kg nicotine base (Sigma Aldrich, St. Louis, MO) and 2 mL/kg. Intraperitoneal (i.p.) injections of 30 or 60 mg/kg vitexin (Cayman Chemical Company, Ann Arbor, MI; Item 15116, Batch: 0500575) or its vehicle (5% DMSO, 5% Tween-80, 90% saline) were given at 3 or 6 mL/kg, respectively.

### Determination of time to habituation and time to peak effect of nicotine

To determine time to habituation, untreated rats were placed in separate 44.5 cm × 33 cm × 38 cm polypropylene spontaneous activity chambers (Sterilite Corporation, Townsend, MA) for 40 min while their locomotor activity was recorded using the Limelight video tracking system (ActiMetrics, Wilmette, IL). After 40 min, rats were given injections (s.c.) of either vehicle (water) or 0.4 mg/kg nicotine, and were placed back into the activity chambers where their activity was recorded for another 60 min (*n* = 7–18).

### Determination of the effect of vitexin on nicotine sensitization

The protocol and data analysis was very similar to that previously published with the exception of the intervention agent (vitexin) (Breivogel and Jamerson 2012). A total of 32 drug-naïve rats were injected s.c. with the pretreatment of either 0.4 mg/kg nicotine or vehicle (water) once per day for four consecutive days at approximately 9:00 am. On the fifth day (test day), the rats (4 at a time) were first put in the spontaneous activity chambers for 30 min to permit habituation. Then each rat was given an i.p. injection of the intervention, vitexin, at 30 or 60 mg/kg or vehicle (5% DMSO, 5% Tween-80, 90% saline) and were then placed back in chambers for another 30 min. At that time, every rat received the challenge dose of 0.4 mg/kg nicotine s.c. and activity was recorded for another 60 min. This produced six treatment groups with the names referring to the 4-day pretreatment and challenge day intervention (*n* in each group): vehicle–vehicle (6), vehicle–30 mg/kg vitexin (4), vehicle–60 mg/kg vitexin (8), nicotine–vehicle (6), nicotine–30 mg/kg vitexin (4) and nicotine–60 mg/kg vitexin (6).

### Determination of the effect of 60 mg/kg vitexin alone on locomotor activity

Rats were given 60 mg/kg vitexin or vehicle (i.p.) and were returned to their home cages for 30 min. Rats were then placed in the spontaneous activity chambers and their activity was recorded for 30 min (*n* = 4 per treatment).

### Data analysis

For all experiments, average velocity in cm/s was calculated for each rat over every 5 min interval by averaging the velocities at each second as provided by Limelight. Figures show the time at the end of each 5-min interval. For all experiments, 2-way ANOVA (treatment × time) with an α of 0.05 was used to determine significant differences between treatment groups and Tukey’s multiple comparisons test identified the time points at which these differences between groups occurred.

## Results

The passion flower extract that was previously shown to antagonize the expression of nicotine locomotor sensitization (Breivogel and Jamerson 2012) was shown to contain a number of flavonoids. The most prevalent were isovitexin, vitexin 2′-*O*-β-d-glucoside (hereafter ‘vitexin-glucoside’) and isovitexin 2′-*O*-β-d-glucoside (hereafter ‘isovitexin-glucoside’). Smaller amounts of orientin and isomers, apiin and isomers and luteolin were also detected (Table 1). *In vitro* screening using agonists for a variety of receptors showed that isovitexin and vitexin-glucoside partially or completely blocked stimulation of [^35^S]GTPγS binding by the serotonin 5-HT_4_ receptor agonist, cisapride and vitexin blocked dopamine D_2_ receptor activation by cabergoline (unpublished observations from our laboratory).

**Table 1.** Results of HPLC-MS analysis of passion flower extract.

Compound	Peak area	*m*/*z*	Retention time (min)	Approximated amount in 5 µL SPE extract
Isovitexin	55,700,000	433.102	5.02	550 ng
Vitexin-glucoside or isovitexin-glucoside	10,600,000	595.1519	0.85	260 ng
	15,400,000	595.1522	1.85	
	Total: 2.60E7			
Orientin or isomer	5,500,000		1.31	100 ng
	4,800,000		1.54	
	Total 1.03E7			
Apiin or isomer	1,330,000	565.1567	0.95	36 ng
	2,320,000	565.1566	1.83	
	Total: 3.65E6			
Luteolin	387,000	287.041	10.85	1.3 ng

**Figure 1.**
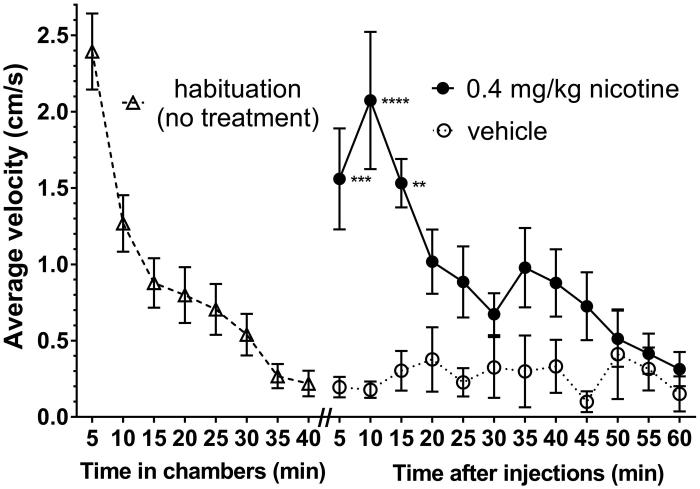
Time to habituation (left) and time course of the acute locomotor effect of nicotine or vehicle (right). To determine time needed to habituate, rats were placed in the spontaneous activity chamber and their activity recorded for 40 min (*n* = 18). At the end of 40 min, rats were injected s.c. with either the vehicle water (*n* = 7) or 0.4 mg/kg nicotine (*n* = 11) and placed back in the chamber for 60 min. Data shown are mean ± SEM for average velocity over each 5-min period. *p < 0.05, **p < 0.01, ***p < 0.001 for nicotine versus vehicle by 2-way ANOVA (time vs. treatment) followed by Tukey’s multiple comparisons test.

The purpose of the first rat experiment was to confirm the length of time it would take for rats to habituate to the activity chambers, and the time course of the locomotor effects of nicotine treatment (Figure 1). The locomotor activity of the untreated rats was initially high for the first 5 min (average velocity of 2.4 cm/s) but steadily subsided to approximately 0.6 cm/s after 30 min and remained at that level or lower over the remaining 30 min. After the subsequent vehicle treatment, locomotor activity remained low with velocities ranging between 0.2 and 0.4 cm/s for the entire 60 min period. During the first 15 min after injection of 0.4 mg/kg s.c. nicotine, rats exhibited significantly greater locomotor activity than vehicle, with values ranging between 1.5 and 2.1 cm/s.

**Figure 2.**
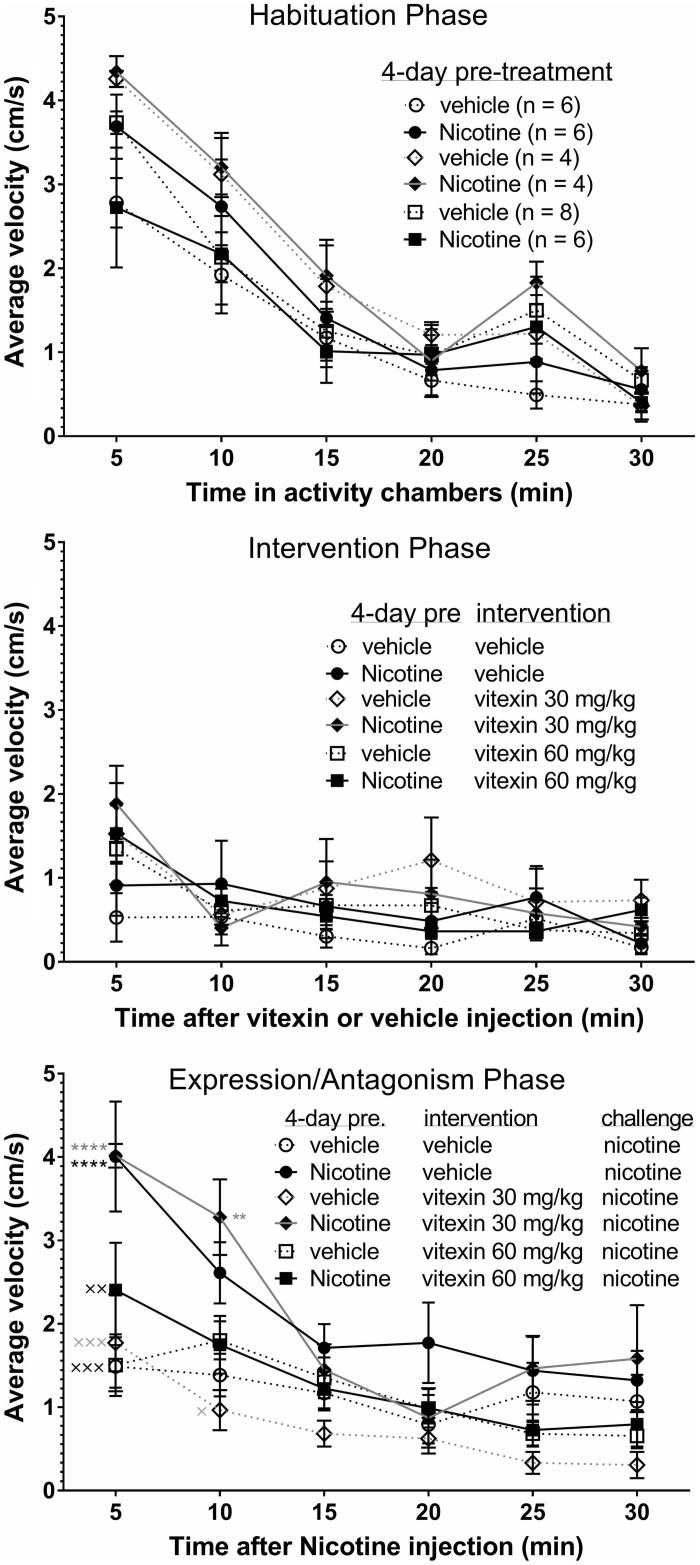
Antagonism of the expression of nicotine locomotor expression by vitexin. Rats were treated once/day for 4 days with 0.4 mg/kg nicotine or vehicle (water). At the end of the habituation phase, each rat was injected with either the intervention, vitexin or vehicle (DMSO: Tween-80: saline) and their activity measured for another 30 min (middle panel). Immediately following, in the expression and antagonism phase, each rats was given the challenge dose of 0.4 mg/kg nicotine s.c. and their activity recorded for another 60 min to assess sensitization and antagonism by vitexin (bottom panel). Only the first 30 min of the last phase is shown since there was little change in activity in any group after the first 15 min and no significant differences between groups after 10 min. Data shown are mean ± SEM for average velocity over each 5-min period. **p < 0.01, ****p < 0.0001 versus vehicle–vehicle–nicotine (control) group, and ^x^*p* < 0.05, ^xx^*p* < 0.01, ^xxx^*p* < 0.001 versus nicotine–vehicle–vehicle (nicotine-sensitized) group by 2-way ANOVA (time vs. treatment) followed by Tukey’s multiple comparisons test.

To assess nicotine sensitization and whether vitexin could antagonize it, rats were given either 0.4 mg/kg nicotine or vehicle once per day for four consecutive days. On the fifth day, locomotor activity was determined during each of the three phases (Figure 2): habituation for 30 min (Figure 2, top panel), intervention of vitexin (30 or 60 mg/kg) or vehicle for 30 min (Figure 2, middle panel), and following the challenge dose of nicotine to assess sensitization and antagonism for 60 min (Figure 2, bottom panel). There were no significant differences between any of the six groups during the first two phases, however, differences were seen during the first 10 min of the third phase. There were no differences between any of the rats pretreated with vehicle on days 1–4, regardless of treatment with vitexin or its vehicle on day 5, and an increase in activity following the nicotine challenge on day 5 demonstrated the acute stimulatory effect of nicotine. Rats treated with nicotine on days 1–4, but not with the intervention of vitexin, showed sensitization to nicotine as evidenced by significantly greater average activity over the first 5 min (*p* < 0.0001) compared to those that were given vehicle on days 1–4 and vehicle as the intervention (average velocities of 4.0 cm/s and 1.5 cm/s, respectively). The activity at 5 and 10 min of the nicotine-sensitized rats that received the pretreatment with 30 mg/kg vitexin was not different from the nicotine-sensitized rats that had the vehicle pretreatment, but was different than rats not sensitized to nicotine (*p* < 0.01 versus vehicle–vehicle group and *p* < 0.05 versus the vehicle–30 mg/kg vitexin group). However, the activity of the nicotine-sensitized rats that received the pretreatment with 60 mg/kg vitexin (nicotine–60 mg/kg vitexin) was significantly lower (*p* < 0.05) than the activity of the nicotine-sensitized rats that had the vehicle intervention (nicotine–vehicle), but not significantly different from any of the un-sensitized groups.

**Figure 3.**
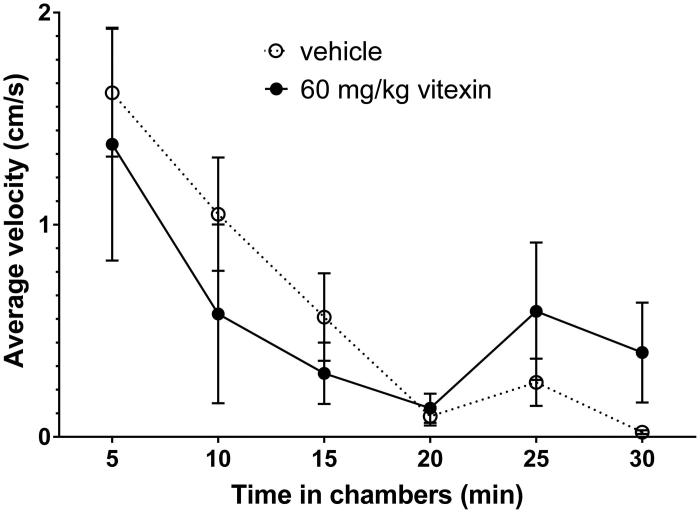
The effect of 60 mg/kg vitexin on rat spontaneous activity. Rats were injected i.p. with either the vehicle (5% DMSO, 5% Tween-80, 90% saline) or 60 mg/kg vitexin and were returned to their home cages for 30 min before being placed in the spontaneous activity chambers and their activity recorded for 30 min. Data shown are mean ± SEM for average velocity over each 5-min period (*n* = 4 per treatment). No significant differences (*p* = 0.374) were observed by 2-way ANOVA (time versus treatment).

It was necessary to confirm that the 60 mg/kg dose of vitexin that antagonized the expression of nicotine locomotor sensitization did not have a nonspecific effect on locomotor activity in non-habituated rats (Figure 3). To that end, activity was assessed after vitexin administration without being allowed to habituate to the activity chambers, but after a delay of 30 min to match the period between the injection of the intervention agent, vitexin and the challenge dose of nicotine in the previous experiments. Rats showed no differences in locomotor activity between vitexin at 60 mg/kg and vehicle (5% DMSO, 5% Tween-80, 90% saline).

## Discussion

In previous studies, passion flower extract demonstrated the ability to attenuate the expression of nicotine locomotor sensitization in rats (Breivogel and Jamerson 2012). Although no vitexin was found in the extract, it is likely that some vitexin-glucoside would be deglycosylated *in vivo* to vitexin (Nemeth et al. 2003; Hostetler et al. 2017). Since dopamine D_2_ receptors are more likely to be involved in the addictive effects of a drug than serotonin 5-HT_4_ receptors, vitexin was considered the flavonoid in the passion flower extract most likely to be responsible for the antagonism of the expression of nicotine locomotor sensitization observed previously. Thus, vitexin was selected for the behavioral experiments of this study.

The results from the present study indicated that 30 min was sufficient for the rats to decrease their spontaneous exploratory behavior, in agreement with previous results (Breivogel and Jamerson 2012). After the habituated rats were treated with nicotine, their locomotor activity increased to a significantly greater degree than those treated with vehicle. This supports the conclusion that the rats were given enough time to habituate and that the dose of nicotine used produced a measurable and statistically significant increase in locomotor activity.

The main goal of the study was to determine if vitexin antagonizes the expression of nicotine locomotor sensitization. Prior to the nicotine challenge dose, nicotine treatments alone did not affect the locomotor activity or habituation time of the rats (Figure 2, top panel). Furthermore, neither dose of vitexin altered the activity of the rats prior to the nicotine challenge, regardless of pretreatment on the first four days (Figure 2, middle panel). These results indicate that the activity of the rats was not affected by the nicotine pretreatments on days 1–4 or by the vitexin at either dose in the first 30 min after administration. Locomotor sensitization to nicotine was seen after four daily injections of nicotine (Figure 2, bottom panel). This was shown by the greater increase in activity in the nicotine–vehicle group compared to the vehicle–vehicle group (or any group that received the 4 day vehicle pretreatment). In the final (antagonism) phase of the experiment, there was no difference between the nicotine–30 mg/kg vitexin group and the nicotine–vehicle group, but there was a significant difference between the nicotine–30 mg/kg vitexin and vehicle–vehicle groups, indicating that the 30 mg/kg dose of vitexin did not affect the expression of nicotine sensitization. However, the nicotine–60 mg/kg vitexin group showed a significant decrease in activity compared to the nicotine–vehicle group. Moreover, there were no differences between the nicotine–60 mg/kg vitexin group and the vehicle–vehicle group (or any group that received the 4-day vehicle pretreatment). This indicated that the 60 mg/kg dose of vitexin selectively antagonized expression of nicotine sensitization.

In the second phase of the antagonism experiment (the intervention phase that began with injection of vitexin or vehicle), it was observed that neither dose had a significant effect on locomotion during the 30 min prior to the nicotine challenge. However, habituation results in very low levels of spontaneous activity, making it difficult to detect more subtle sedative effects. The final experiment was a control to determine if vitexin had a sedative effect on locomotor activity on its own during the time that nicotine was showing locomotor sensitization. Thus, it was important to test the acute locomotor effects of vitexin under conditions where the rats had not been habituated to the testing environment and spontaneous activity was relatively high. The 60 mg/kg vitexin dose did not affect locomotion compared to vehicle in this experiment either. This indicates that the lower levels of locomotion observed in the antagonism of the expression of nicotine locomotor sensitization experiment (5–10 min after nicotine and 35–40 min after vitexin administration) were not caused by an acute sedative effect of vitexin. These data support the conclusion that the effect of 60 mg/kg vitexin represents specific antagonism of the expression of nicotine locomotor sensitization.

## Conclusions

Previous work has demonstrated that a commercial extract of passion flower antagonized the expression of nicotine locomotor sensitization in rats (Breivogel and Jamerson 2012). The results from this study suggest that vitexin is an active ingredient in passion flower responsible for its effect on nicotine locomotor sensitization, but does not rule out the possibility that other compounds in passion flower contribute to this effect or have a similar effect. The medication varenicline was validated as a potential drug for smoking cessation using this same protocol including the strain of rats (Zaniewska et al. 2008). Following subsequent clinical trials, varenicline was approved based on demonstrated increases in the chances of quitting tobacco smoking (Gonzales et al. 2006). Therefore, vitexin shows potential as a new pharmacotherapy to aid in smoking cessation.
